# Lipid homeostasis in diabetic kidney disease

**DOI:** 10.7150/ijbs.95216

**Published:** 2024-07-02

**Authors:** Ying Wang, Tongtong Liu, Yun Wu, Lin Wang, Shaowei Ding, Baoluo Hou, Hailing Zhao, Weijing Liu, Ping Li

**Affiliations:** 1 China-Japan Friendship Hospital, Institute of Medical Science, Beijing, China.; 2 Key Laboratory of Chinese Internal Medicine of Ministry of Education and Beijing, Dongzhimen Hospital Affiliated to Beijing University of Chinese Medicine, Beijing, China.; 3 Guang'anmen Hospital, China Academy of Chinese Medical Sciences, Beijing, China.

**Keywords:** Diabetic kidney disease, Lipid homeostasis, Lipotoxicity, Autophagy.

## Abstract

Lipid homeostasis is crucial for proper cellular and systemic functions. A growing number of studies confirm the importance of lipid homeostasis in diabetic kidney disease (DKD). Lipotoxicity caused by imbalance in renal lipid homeostasis can further exasperate renal injury. Large lipid deposits and lipid droplet accumulation are present in the kidneys of DKD patients. Autophagy plays a critical role in DKD lipid homeostasis and is involved in the regulation of lipid content. Inhibition or reduction of autophagy can lead to lipid accumulation, which in turn further affects autophagy. Lipophagy selectively recognizes and degrades lipids and helps to regulate cellular lipid metabolism and maintain intracellular lipid homeostasis. Therefore, we provide a systematic review of fatty acid, cholesterol, and sphingolipid metabolism, and discuss the responses of different renal intrinsic cells to imbalances in lipid homeostasis. Finally, we discuss the mechanism by which autophagy, especially lipophagy, maintains lipid homeostasis to support the development of new DKD drugs targeting lipid homeostasis.

## Introduction

Diabetic kidney disease (DKD) is the most common complication of diabetes mellitus (DM) and the leading cause of end-stage renal disease (ESRD) 1, and it is also one of the fastest growing causes of chronic kidney disease (CKD) morbidity and mortality 2. DKD is characterized by the accumulation of collagen, fibronectin, and other extracellular matrix (ECM) proteins. Such accumulation leads to inflammation induced by tubulointerstitial fibrosis, glomerular plasma membrane hypertrophy and dilatation, thickening of the glomerular basement membrane, loss of podocyte foot processes, and infiltration of monocytes and macrophages 3. Disorders of lipid metabolism are associated with renal insufficiency and the pathological features of DKD 4. Increasing evidence has shown that imbalances in lipid homeostasis and lipotoxicity can lead to kidney injury in DKD 5. Lipid accumulation in the kidney is related to glomerulosclerosis and tubulointerstitial damage 6, 7. Significant lipid deposition and lipid droplet (LD) accumulation are found in the kidneys of patients with DKD 8. Lipid deposition has been reported in diabetic glomerulosclerosis 8, 9. Lipotoxicity due to fatty acid (FA) deposition and renal tubulointerstitial fibrosis characterized by epithelial-mesenchymal transition (EMT) are hallmarks of DKD 10. There are differences in the lipid metabolic profile in various stages of DKD 11. Macroalbuminuria is associated with elevated total cholesterol levels in DKD 12. Dyslipidemia, which is characterized by high levels of triglyceride-rich lipoproteins, low levels of high-density lipoprotein-cholesterol (HDL-C), and high levels of oxidized low-density lipoprotein (oxLDL), accelerates DKD progression 13, 14. Therefore, close crosstalk occurs between lipid metabolism and DKD.

Lipids are the primary source of energy for the kidney, which is a highly energy-intensive organ 15. The total lipid content of the kidney in healthy individuals is about 3% of its wet weight 16, with more than half of the lipid content being phospholipids, about one-fifth being triglycerides, and about one-tenth being nonesterified FAs (NEFAs). In humans, the level of kidney-extracted FAs is linearly related to plasma FA concentrations 16. The levels of cholesterol, phospholipids, triglycerides, FAs, and sphingolipids are altered in DKD, and their accumulation in the kidney is associated with DKD pathogenesis 17. FA oxidation (FAO) can be reduced by disrupting the balance between FA synthesis, intake, and consumption, thereby affecting renal lipid metabolism and leading to intracellular lipid accumulation 18. Additionally, dyslipidemia alters lipid homeostasis by causing apoptosis of podocytes and endothelial cells, macrophage activation, and mesangial matrix hyperplasia, as well as increasing lipoprotein receptor-mediated cholesterol uptake, inhibiting ATP-binding cassette protein 1-mediated cholesterol efflux, and impairing cholesterol synthesis in peripheral cells 19. However, the renoprotective effect of lipid-lowering therapy remains controversial 20, 21.

Here, we describe the major regulatory molecules of renal lipid metabolism homeostasis, discuss lipid metabolism in various types of renal intrinsic cells, and summarize the current state of research on the role of autophagy in lipid metabolism. As mentioned earlier, the imbalance of lipid homeostasis and lipotoxicity can aggravate DKD progression. This article aims to support the development of new DKD drugs targeting lipid homeostasis.

## Renal lipid homeostasis

The mechanisms of renal lipid accumulation may differ among different causes of chronic kidney disease. In glomerulonephritis, inflammation can disrupt normal renal lipid metabolism. However, renal lipid accumulation in DKD is mainly driven by increased glucose and fatty acids levels owing to insulin resistance 22. Under normal conditions, the uptake, synthesis, and oxidation or efflux of FAs in renal cells are regulated by a series of transcription factors to achieve a balanced and coordinated system to avoid lipid accumulation in the cells 8, 23. FA synthase (FAS) and acetyl-CoA carboxylase (ACC) catalyze FA synthesis, whereas stearoyl-CoA desaturase 1 is a rate-limiting enzyme that converts saturated FAs to monounsaturated FAs. FAs are translocated to the mitochondria and degraded (through β-oxidation) by carnitine palmitoyltransferase 1 (CPT1) and acyl-CoA oxidase 8.

**Figure 1:**
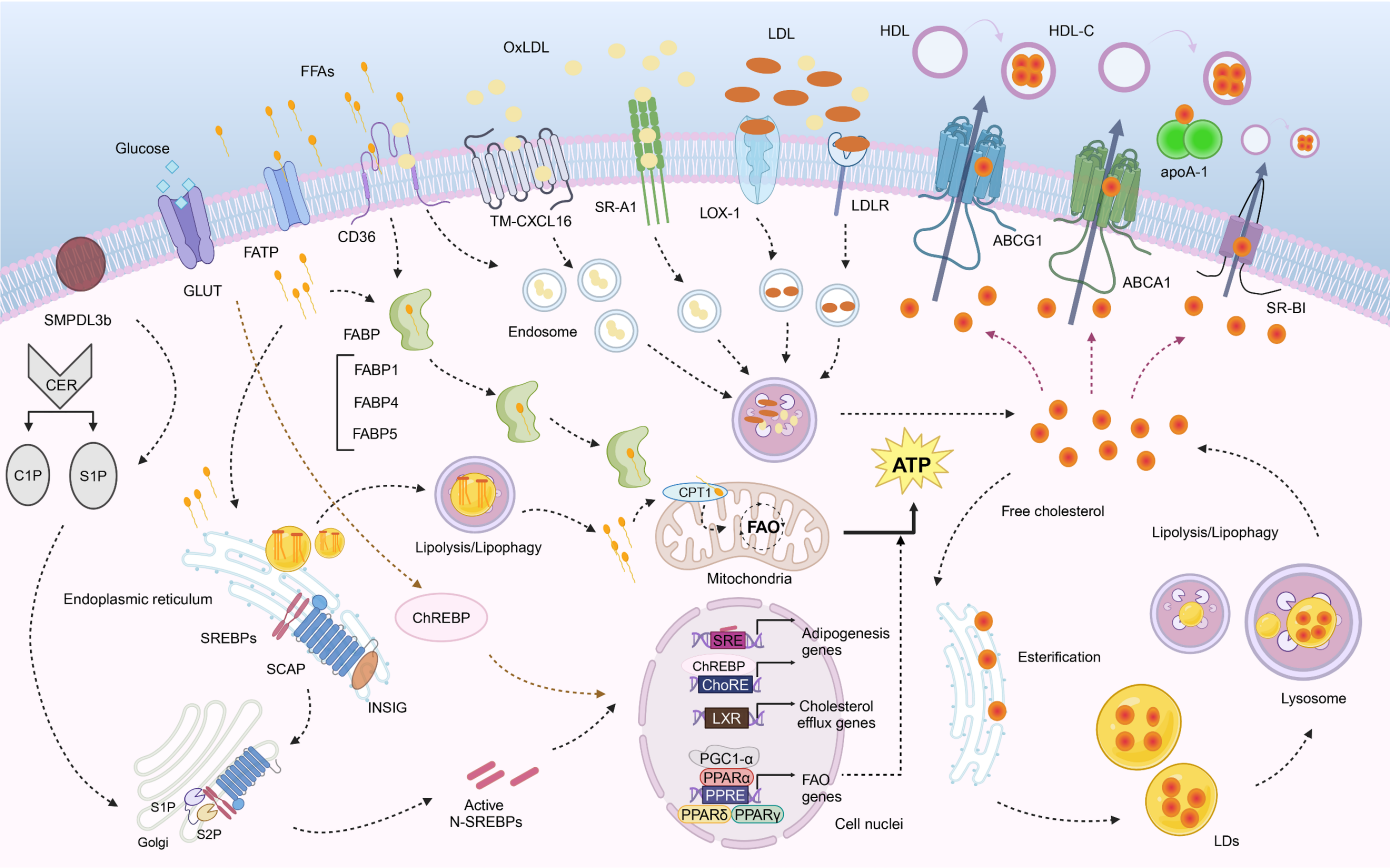
** Fatty acid, cholesterol and sphingolipid metabolism.** (1) FAs are taken up by cells through CD36, CXCL16, and FATP, and transported to mitochondria via FABP. They also synthesize TGs in the endoplasmic reticulum, which are stored as LDs. These LDs can be decomposed by lysosomes or undergo lipophagy (not shown in the figure). CPT1 on the outer membrane of the mitochondria serves as a carrier to control FA β-oxidation of medium- and long-chain FAs. The oxidation-related genes are regulated by PPAR and PGC-1α. SREBPs and ChREBPs can affect lipid synthesis. (2) Cholesterol influx mainly depends on SR-A1, CD36, LOX-1, LDLR. Cholesterol production is primarily regulated by SREBP-2 and can be activated in the Golgi apparatus. Free cholesterol can be esterified in the endoplasmic reticulum and stored as LDs that may undergo decomposition by lysosomes or lipophagy (not shown in the figure). Cholesterol efflux is mainly mediated by ABCA1, ABCG1, and SR-BI under regulation from LXR. (3) Sphingolipids include CER, C1P and S1P, etc.,while SMPDL3b regulates cell membrane fluidity associated with podocyte function.

Lipotoxicity is a metabolic condition caused by the intracellular accumulation of toxic lipid intermediates in nonadipose tissues, thus resulting in cellular dysfunction and potential cell death (lipoapoptosis) 24. FAs are essential for cell structure, function, and signaling. In the blood, free FAs (FFAs) are transported by serum albumin as complex lipoproteins 25. In kidney disease patients, oxidative modification of HDL and LDL particles occurs, leading to the formation of small lipoproteins and enhanced synthesis of oxLDL 19. FFAs produced by adipocytes or released by extracellular lipases are transported into cells by membrane-associated proteins that include scavenger receptor B2 (SR-B2; also known as differentiation antigen 36 [CD36]), FA transporter proteins (FATPs), and FA-binding proteins (FABPs) or by passive diffusion 26. The discussion on FA uptake, transport, oxidation, and synthesis below also includes cholesterol metabolism and sphingolipid metabolism (**Fig. 1**).

### Fatty acid metabolism

#### Fatty acid uptake and transport

FAs serve as an important source of energy for the body. FAs are taken up and transported to mitochondria for oxidation. This process involves molecules that include CD36, CXCL16, FATP, and FABP. FAs are taken up by proximal tubular cells mainly via CD36 10. CD36 is a transmembrane glycoprotein that transports FAs into cells and is expressed primarily in renal tubular epithelial cells (TECs), podocytes, and mesangial cells (MCs) in the kidney, where it can act as a receptor for long-chain FAs, and oxidized lipids and play a role in lipid accumulation, inflammatory signaling, and renal fibrosis 27. However, there is evidence that the chemokine CXCL16, but not CD36, is the major scavenger receptor mediating oxLDL uptake in human podocytes 28, 29. OxLDL downregulates integrin α3, increases fibronectin production, and induces reactive oxygen species (ROS) production in human podocytes 29. FATPs are transmembrane proteins involved in FA uptake and activation. The FATP family consists of six tissue-specific isoforms, of which the kidney predominantly expresses FATP1, FATP2, and FATP4 30. FATPs can catalyze the formation of CoA derivatives with long-chain and ultra-long-chain FAs, bile acids, and bile acid precursors as substrates 31. FATP2, one of the primary FA transporters expressed in renal TECs, induces reprogramming of lipid metabolism, including abnormal FA uptake and FAO defects, triggering renal interstitial fibrosis, which is closely associated with decreased renal function 32. FABPs are intracellular lipid chaperones involved in the regulation of intracellular lipid transport and responses, and they are related to metabolic and inflammatory pathways 33. FABPs are small, water-soluble proteins that bind to long-chain FAs and other bioactive ligands to promote intracellular localization 34. FABP1 is expressed in renal proximal tubular cells and is released into the urine in response to hypoxia caused by reduced peritubular capillary blood flow. FABP2 is responsible for the transport of FFAs in intestinal endothelial cells. Both FABP1 and FABP2 are biomarkers of DKD 35. Urinary liver-type FA-binding protein (L-FABP) indicates the extent of tubulointerstitial damage 36, and it is an invaluable marker of DKD progression 37.

#### Fatty acid oxidation

FAO, the catabolic process by which FAs are broken down into acetyl-CoA, is the preferred energy source for higher metabolizing cells and takes place primarily in mitochondria 18. CPT1A is a carrier of medium- and long-chain FAs into mitochondria, binds FAs to carnitine, and is a key rate-limiting enzyme for FAO 38. In three models of renal fibrosis (unilateral ureteral obstruction, folic acid nephropathy, and adenine-induced nephrotoxicity), the extent of renal fibrosis was reduced in CPT1A knockin (CPT1A-KI) mice, and a protective effect against fibrosis was seen following inducing CPT1A overexpression 39. Peroxisome proliferator-activated receptor (PPAR) and PPAR-γ coactivator-1α (PGC-1α) are key transcription factors that regulate the expression of FA uptake- and oxidation-related proteins 40, 41. PPAR belongs to the type II nuclear hormone receptor superfamily, which is organized into three isoforms: PPAR-α, PPAR-β/δ, and PPAR-γ. It can act as a transcription factor that binds to response elements within the promoters of genes related to glycolipid metabolism 42. PPAR-α and PPAR-β/δ regulate FAO, whereas PPAR-γ is more closely linked to adipogenesis and lipid storage 43. PPAR-α promotes FAO and oxidative phosphorylation 44. In the DKD model, reduced PPAR-α and PPAR-δ expression can lead to decreased FAO 41 and is associated with a decreased estimated glomerular filtration rate (eGFR) 8. PPAR-γ participates in the maintenance of renal metabolic homeostasis, and PPAR-γ inhibition leads to renal tubular hypertrophy, tubulointerstitial fibrosis, and impaired renal function 45. PGC-1α participates in cellular lipid metabolism and energy regulation by interacting with PPAR to influence the expression of genes related to lipid synthesis and transport 46. FABP4, one of the isoforms of FABP, is able to translocate FAs and downregulate its target gene, PPARG, encoding PPAR-γ 47.

#### Lipogenesis

Enhanced activation of adipogenic genes will promote glomerular and tubular lipid deposition. In the DKD model, increased renal lipid levels were associated with increased expression of renal sterol regulatory element-binding proteins (SREBPs) and carbohydrate response element-binding proteins (ChREBPs) 41, 48-50. SREBPs (SREBP1a, SREBP1c, and SREBP2) belong to the family of membrane-bound transcription factors involved in the regulation of lipid synthesis, with SREBP1a being responsible for overall lipid synthesis, SREBP1c being responsible for FA and triglyceride synthesis, and SREBP2 specifically controlling steroidogenesis 51. In DKD patients, increased expression of SREBP1 and SREBP2 causes renal lipid deposition, lipotoxicity, and fibrosis 23. In unilateral ureteral obstruction rats, blockade of SREBP1/2 signaling using SREBP inhibitors significantly attenuates renal inflammation, necrosis, and fibrosis by affecting lipogenesis and transforming growth factor-β1 (TGF-β1) expression 52. Junctional adhesion molecule-like protein (JAML) regulates podocyte lipid metabolism through SIRT1-mediated SREBP1 signaling 53. Farnesoid X receptor (FXR) agonists modulate SREBP1 expression, lipid metabolism, and the expression of renal profibrotic growth factor, proinflammatory cytokines, and oxidative stress enzymes and reduce glomerulosclerosis, tubulointerstitial fibrosis, and proteinuria. ChREBP is a primary mediator of the action of glucose on the expression of adipogenic genes and a key determinant of lipid synthesis *in vivo* 54. ChREBP regulates the expression of ACC and FAS and regulates adipogenesis by inducing expression of the glycolytic enzyme pyruvate kinase 1 55.

### Cholesterol metabolism

In addition to FA metabolism, dysregulation of renal cellular cholesterol metabolism has also been linked to lipotoxicity and lipid accumulation in DM, caused by alterations in cholesterol uptake, intracellular synthesis, esterification, and efflux 56. Cholesterol influx into cells is mediated by several independent receptors, including the class A scavenger receptor CD36, lectin-like oxLDL receptor-1 57, and the LDL receptor (LDLR) 28. A highly significant correlation was found between increased expression of LDLR, oxLDL, acetylated LDL (acLDL), and CD36 and progression of DKD and worsening of eGFR 8. SREBP cleavage-activating protein (SCAP) is thought to be a chaperone for SREBP2, which is transported from the endoplasmic reticulum (ER) to the Golgi apparatus for activation via proteolytic cleavage 58. 3-Hydroxy-3-methylglutaryl-CoA (HMG-CoA) reductase is involved in the maintenance of cholesterol synthesis, which involves SREBP2 59. In the kidneys of diabetic rats, LD accumulation and increases in HMG-CoA reductase, LDLR, SREBP2, and SCAP levels were observed 60. The preprotein convertase chymotrypsin 9 (PCSK9) is a crucial protein in the regulation of lipid metabolism in which SREBP2 is involved 61. PCSK9 binds to surface LDLR, leading to its degradation and subsequent elevation of plasma LDL-C levels 62, 63. In a clinical study, mean serum PCSK9 concentrations were higher in CKD patients than in controls 64.

Cholesterol efflux is primarily mediated by ATP-binding cassette transporter proteins, including ATP-binding cassette transporter A1 (ABCA1) and ATP-binding cassette transporter G1 (ABCG1), and SR-BI 65, 66. ABCA1 promotes cholesterol efflux from cells and inhibits inflammatory responses and is highly expressed in the kidney 67. Liver X receptor alpha (LXRα) and LXRβ are members of the nuclear receptor family that crucially regulate cholesterol homeostasis. In DKD, the expression of the cholesterol efflux genes ABCA1, ABCG1, and apoE was reduced, and the expression of nuclear receptor LXRα, which regulates cholesterol efflux genes, was also downregulated 68. ABCA1 deficiency in glomerular endothelial cells exacerbated renal lipid deposition and renal injury in type 2 DM mice, as evidenced by elevated creatinine levels, more severe proteinuria, and more pronounced dilatation of the thylakoid matrix, pedunculopapillary fusion, renal inflammatory injury, and cell death 65. ABCA1 deficiency is associated with cardiolipin-driven mitochondrial dysfunction, ultimately leading to DKD 69. PPAR-γ also plays an essential role in regulating the uptake of oxLDL by upregulating CD36 expression and LXR-ABCA1-mediated cholesterol efflux 70, 71. Apolipoprotein L1 (APOL1) is a secreted HDL that co-localizes with APOA1 in HDL particles, which promotes cholesterol efflux from cells 72.

### Sphingolipid metabolism

Sphingolipids are defined as compounds containing long-chain bases or sphingamines. Sphingolipids have also been shown to play an essential role in the onset and development of DKD. Sphingolipids and their metabolites, including ceramide (CER), sphingosine, CER-1-phosphate (C1P), and sphingosine-1-phosphate (S1P), as biologically active signaling molecules, perform vital actions in intracellular signaling and may be involved in the regulation of apoptosis, autophagy, inflammation, immunity, and membrane fluidity 73. Following 4 days of streptozotocin (STZ) treatment, the levels of neutral ceramidase, SK activity, and S1P were significantly elevated in isolated glomeruli of rats, thus suggesting that S1P may be involved in the early glomerular proliferative response in DKD 74. Elevated albuminuria and increased connective tissue growth factor expression were found in SK-1 knockout mice as compared to wild-type C57BL/6 mice 75. CER is a biologically active sphingolipid that is a substrate for the production of C1P and S1P 76. Increased CER production due to upregulation of serine palmitoyltransferase, a key enzyme involved in *de novo* CER synthesis, is associated with increased apoptosis in STZ-induced DKD in renal TECs and microvascular endothelial cells 77.

Sphingolipids, especially sphingolipids and sphingolipid sugars, form aggregates in various regions with cholesterol, frequently referred to as lipid rafts 78. Sphingomyelin phosphodiesterase acid-like 3b (SMPDL3b) is a lipid raft sphingomyelinase that modifies plasma lipid composition, regulates intracellular inflammatory pathways, and controls the ability of circulating factors to affect podocyte function and survival 20. SMPDL3b overdose resulted in reduced C1P levels and impaired insulin-mediated prosurvival signaling pathways in cultured human podocytes *in vitro* and in the renal cortex of DKD mice *in vivo* 79. In db/db mice, podocyte-specific SMPDL3b deficiency restores renal cortical C1P content, which in turn prevents DKD 79. The potential role of glycosphingolipid accumulation in DKD suggests that hyperglycemia is associated with enhanced synthesis of glucosylceramide and ganglioside GM3, thus ultimately leading to renal hypertrophy in STZ-induced diabetic rats 80.

## Lipid homeostasis imbalance and DKD progression

**Figure 2:**
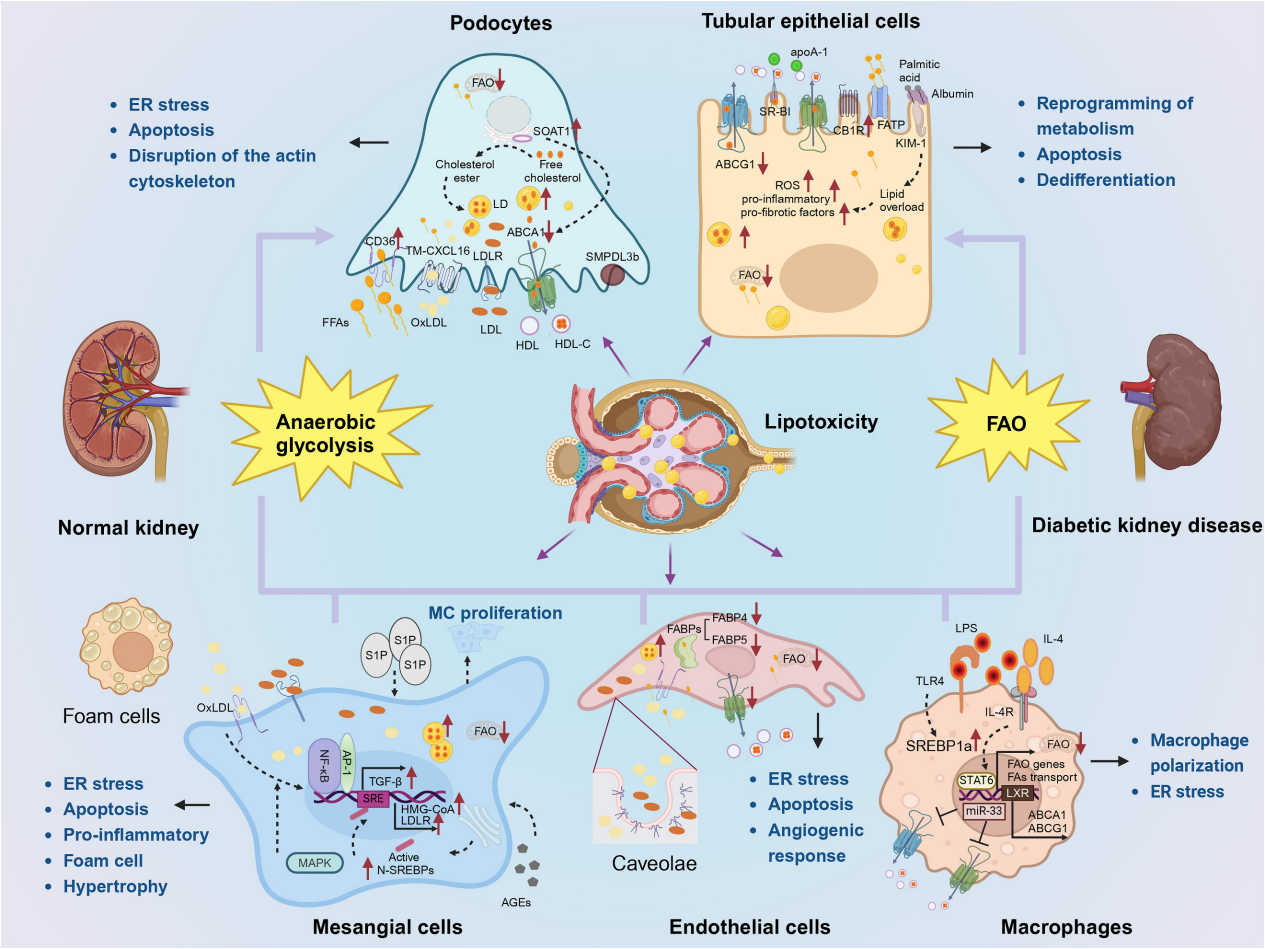
** Lipid metabolism in renal cells in DKD.** (1) Podocytes primarily rely on anaerobic glycolysis as their main energy source, with lipid metabolism being mainly regulated by CD36, LDLR, CXCL16, and ABCA1. An imbalance in lipid homeostasis can result in endoplasmic reticulum stress, apoptosis, and disruption of the actin cytoskeleton. (2) Renal tubular epithelial cells predominantly utilize FAO as their primary energy source, with lipid metabolism being mainly regulated by ABCA1, ABCG1, SRBI, CB1R, FATP, and KIM-1. An imbalance in lipid homeostasis can lead to metabolic reprogramming, apoptosis, and increased dedifferentiation. (3) Lipid accumulation in mesangial cells is primarily associated with LDL and oxLDL uptake while MAPK, NF-κB, and AP-1 are involved in regulation. An imbalance of lipid homeostasis will lead to endoplasmic reticulum stress, apoptosis, inflammation, foam cells, mesangial cell proliferation and hypertrophy. (4) Caveolae play a crucial role for endothelial cells' uptake of lipids. Lipid metabolism is mainly influenced by FABP, ABCA1, and sphingolipid metabolites. An imbalance of lipid homeostasis will cause endoplasmic reticulum stress, apoptosis, and affect angiogenic responses. (5) Macrophage depolarization is closely related to lipid homeostasis.

Lipid molecule signaling imbalances and lipid accumulation in nonadipose tissues can negatively affect homeostasis 81. There is a growing consensus that ectopic lipids (accumulation of lipids in nonadipose tissue) are associated with structural and functional changes in MCs, podocytes, and proximal renal tubule cells 82. Crosstalk between cells in kidney homeostasis plays a vital role 83. Dysregulation of lipid metabolism in renal intrinsic cells is able to contribute more directly to renal lipotoxicity as opposed to dyslipidemia (**Fig. 2**).

### Lipid metabolism in podocytes

Podocytes are essential for maintaining a normally functioning glomerular filtration barrier. Loss of podocytes leads to a reduction in glomerular basement membrane coverage, which initially manifests as albuminuria and eventually as glomerulosclerosis 84. Podocytes are sensitive to lipid accumulation 69, 85. Lipid accumulation in podocytes is a major determinant of proteinuric nephropathies, including obesity-associated nephropathy, DKD, and focal segmental glomerulosclerosis 53, 86. Lipotoxicity and lipid accumulation leading to podocyte damage and apoptosis in DKD patients are characteristic features of DKD, and podocytopenia is an independent predictor of DKD progression 7, 50. Anaerobic glycolysis and the fermentation of glucose to lactate are key processes providing energy for podocytes 87. Saturated FFAs lead to ER stress and apoptosis in podocytes 88, 89, whereas stearoyl-CoA desaturase-1 upregulation attenuates ER stress and podocyte apoptosis 90. Enhanced FFA uptake by podocytes is mediated by increased expression of the C36 scavenger receptor, as well as decreased β-oxidation and intracellular lipid accumulation 28. Dysregulated transport and oxidation of FFAs, coupled with impaired antioxidant responses, cause structural damage to the podocyte, leading to early DKD glomerulopathy 28. LDLR is the primary receptor mediating lipid uptake in podocytes, and high glucose dysregulates feedback regulation of the LDLR pathway 20. Treatment of human podocytes with serum from patients with DKD resulted in more cholesterol accumulation compared with human podocytes exposed to serum from diabetic patients (but not DKD) at the same total cholesterol, HDL-C, and LDL concentrations 91. Podocyte survival and the integrity of the actin cytoskeleton are compromised following exposure to oxLDL 92, 93. Elevated tumor necrosis factor (TNF) levels promote free cholesterol-dependent podocyte depletion via ABCA1-mediated reduction of cholesterol efflux and cholesterol esterification by cholesterol-O-acyltransferase 1 (SOAT1) 94, 95. Inhibition of SOAT1 in human podocytes reduced lipotoxic injury while increasing ABCA1 expression and ABCA1-mediated cholesterol efflux 96. However, *in vitro* knockdown of ABCA1 in podocytes and *in vivo* podocyte-specific deletion of ABCA1 were insufficient to cause podocyte apoptosis and glomerular injury, respectively 94. SMPDL3b expression is increased in glomeruli from DKD patients and human podocytes treated with DKD serum, which makes podocytes more susceptible to apoptosis through suPAR 97. Podocin is a slit membrane protein in podocytes. Notably, MEC-2 and podocin bind and collect cholesterol to organize the lipid microenvironment of related ion channel complexes. Cholesterol interaction also regulates the glomerular filtration barrier ion channel activity 98, 99. Cholesterol can also enhance inflammatory effects by directly activating inflammasomes, inducing lysosomal damage, and causing toll-like receptor (TLR) localization in lipid rafts 100. SMPDL3b is also involved in TLR33-mediated inflammatory activation 101. In turn, inflammation can upregulate the expression of LDLR, SCAP, and SREBP-2^102^. Inflammation reduction can also be achieved through endogenous specialized solubilizing lipid mediators (SPMs) and branched-chain fatty acid esters of hydroxy fatty acids 103, 104. Therefore, lipids and inflammation can interact and regulate each other.

### Lipid metabolism in renal tubular epithelial cells

The presence of lipid deposits in diabetic TECs was initially described in 1936^105^. FAs are the primary energy source for TECs 15, 18. Therefore, FAO in lipid metabolism, in particular, is the primary pathway by which renal tubulointerstitial fibrosis affects TECs 106. Inhibition of FAO in TECs causes ATP depletion, cell death, dedifferentiation, and intracellular lipid deposition 18.

Decreased FAO in TECs leads to reprogramming of their metabolism, which in turn results in increased apoptosis and dedifferentiation 18. Lipotoxic manifestations, including ROS, release of proinflammatory and profibrotic factors, and apoptosis, can occur as a result of TEC lipid overload due to excessive dietary intake or dysfunctional lipid depletion or degradation 107, 108. In a DKD model, tubule-specific deletion of Pacs2, which encodes a protein associated with lipid metabolism, resulted in severe tubular injury accompanied by increased lipid synthesis and uptake and decreased cholesterol efflux 109. ABCA1, ABCG1, and SR-B1 are expressed in human renal MCs and proximal TECs, all of which mediate cholesterol efflux to ApoA1 and HDL 110. Albumin itself is not cytotoxic to proximal tubules, but NEFAs bound to albumin trigger apoptosis in proximal tubules 111, 112. In contrast with adipocytes, which have large stores of intracellular LDs under physiological conditions, TECs contain limited LDs to maintain their energy homeostasis 113. LDs and LD-associated proteins protect against FA-bound albumin-induced apoptosis by sequestering FFAs 57. FATP2 is a major apical proximal renal tubule NEFA transporter protein that regulates apoptosis in proximal TECs 114. Further, FATP2 deficiency does not completely eliminate FA uptake in the proximal renal tubule 114. Among the FA transport proteins that are not members of the FATP family, kidney injury molecule-1 (KIM-1) is expressed within the proximal tubule membrane and acts as a scavenging receptor for oxidized lipoproteins and apoptotic cells by recognizing exogenous phosphatidylserine 115. DKD mouse models and *in vitro* studies have shown that KIM-1 mediates proximal tubule uptake of albumin-bound palmitate 116. In proximal tubules, palmitate induced proximal tubular cannabinoid expression and enhanced apoptosis, thus suggesting that cannabinoids may mediate proximal tubular lipotoxicity 117. Specific deletion of cannabinoid receptor 1 in renal proximal tubule cells, although not protecting mice from obesity, significantly attenuates obesity-induced renal lipid accumulation and renal dysfunction, injury, inflammation, and fibrosis 118.

### Lipid metabolism in mesangial cells

Prior studies have suggested that MCs exhibit specific binding and uptake of LDL 119 and that lipid accumulation in MCs may be caused by receptor-mediated endocytosis of LDL particles 120. The mesangial matrix has a high capacity to bind LDL in a nonsaturated manner and to modify LDL (by glycation or oxidation) 121. oxLDL induces the expression of activator of transcription factor 1 (AP-1) in rat MCs 122, which regulates TGF-β gene expression through the AP-1-binding site 123, and nuclear factor-κB (NF-κB) is also involved in oxLDL-induced enhancement of ACC 124. Excess oxLDL, especially lipid peroxides and lysophospholipids of oxLDL, may exert cytotoxic effects on MCs, epithelial, and endothelial cells, thereby contributing to a vicious cycle of cellular damage and sclerosis 121. Some oxLDL can be absorbed by scavenger receptors on MCs and mononuclear macrophages, leading to foam cell formation 121. oxHDL enhances the proinflammatory properties of MCs in part through CD36 and LDLR-1, and the mitogen-activated protein kinase (MAPK) and NF-κB pathways are also involved in this process 125. Lipotoxicity can induce the protein expression of arginine methyltransferase 1 to promote ER stress-mediated apoptosis of MCs 126. In MCs, exposure to advanced glycation end products (AGEs) leads to increased SCAP translocation and transport of SREBP2 to the Golgi apparatus, which in turn leads to mesangial foam cell formation through activation of proteolytic cleavage through enhanced transcription of HMG-CoA and LDLR 59. In experimental models of DKD, inhibition of glucosylceramide synthase reversed MC hypertrophy by reducing high glucose-induced phosphorylation of SMAD3 and Akt, alleviating fibrosis, and reducing the expression of ECM proteins 127. S1P stimulates MC proliferation 128, and exposure to the MC matrix induces monocyte differentiation toward a macrophage-like phenotype and promotes LDL oxidation, thereby transforming this lipoprotein into scavenger receptor ligands associated with foam cell formation in the mesangium 129. Insulin-like growth factor-1 induces lipid accumulation in MCs, reducing their ability to respond to specific migratory and contractile stimuli 130.

### Lipid metabolism in endothelial cells

The endothelium is a thin monolayer of cells covering the luminal surface of the vessel wall; it forms a barrier between blood and surrounding tissues and plays an active role in vascular function and homeostasis 131. Caveolae consist of 50- to 100-nm apical plasma membrane invaginations and are important mediators of endothelial endocytosis, endocytosis, lipid homeostasis, and signaling in endothelial cells 132; however, high levels of circulating oxLDL affect caveolae lipid composition and/or function 131. Circulating LDL is absorbed by endothelial cells through receptor-mediated endocytosis or endocytosis 131. In contrast, the only way for LDL to cross the endothelium is through caveolae-mediated endocytosis 131. FABP is closely related to endothelial cells. Cellular aging and oxidative stress induce FABP4 expression in microvascular endothelial cells 133, which can enhance the angiogenic response of endothelial cells, including proliferation, migration, and survival 134. FABP4 generally has higher affinity and selectivity for long-chain FAs than albumin 135. FABP5 deficiency leads to endothelial cell proliferation and chemotactic migration 134. ABCA1 deficiency in glomerular endothelial cells exacerbates renal lipid deposition and renal injury in type 2 diabetic mice 65. ABCA1 overexpression can enhance cholesterol efflux or inhibit ER stress *in vitro* and significantly protect glomerular endothelial injury stimulated by high glucose and high cholesterol 65. Sphingolipid metabolites and related enzymes are closely related to the apoptosis, senescence, oxidative stress, and other activities of endothelial cells and affect the regulatory function of endothelial cells in the stress response, angiogenesis, and the inflammatory response 136.

### Lipid metabolism in macrophages

Macrophages are cells of the innate immune system, which can be polarized into M1 macrophages (proinflammatory role) and M2 macrophages (anti-inflammatory role) depending on the type of stimulus 137. Macrophages can maintain tissue homeostasis, induce immune responses, and participate in tissue repair 138, 139. Studies have shown that lipid synthesis is related to macrophage function. Following lipopolysaccharide stimulation of TLR4, macrophages can increase *de novo* lipogenesis and also activate SREBP1a expression 140-142. Macrophages can also increase the uptake of FFAs and lipoproteins following exposure to inflammatory stimuli 143, 144. IL-4 activates macrophages, and FAMIN proteins link *de novo* lipogenesis to FAO through an apparent “substrate cycle” 141. IL-4 signaling activates signal transducer and activator of transcription 6 (STAT6), which promotes lipid transport, FAO, and mitochondrial biogenesis 145. Macrophage polarization is affected by lipids, which also play an influential role in the control of macrophage function. The accumulation of lipids and inflammatory cytokines can jointly induce ER stress in macrophages 146. Lipotoxic TEC-derived extracellular vesicles (EVs) induce the expression and release of proinflammatory cytokines such as IL-1β and TNF-α in macrophages and the release of macrophage-derived EVs 147. LDs are involved in cellular FA homeostasis and the regulation of macrophage function 145. Deficiency of enzymes involved in FA esterification, including diacylglycerol acyltransferase-1 (DGAT1), increases the proinflammatory response of macrophages 148. Adipose triglyceride lipase (ATGL) is one of the enzymes involved in the breakdown of triglycerides in macrophage LDs, and deficiency of lipases such as ATGL attenuates the expression of proinflammatory genes (such as IL6) and favors the activation of anti-inflammatory macrophages 149. Monoacylglycerol lipase is a lipase that decomposes monoacylglycerol into FFAs and glycerol and is involved in macrophage autophagy and inflammation 150. Inhibition of lysosomal acid lipase (LAL) resulted in M2-type macrophage polarization and reduced mitochondrial oxidative respiration, suggesting that lipolysis is also required for macrophage polarization 151. PPAR-γ is also involved in the suppression of macrophage proinflammatory responses 152. miR-33 is a microRNA involved in SREBP signaling. In macrophages, miR-33 can regulate cholesterol homeostasis by inhibiting the expression of genes encoding ABCA1 and ABCG1^153^. LXR regulates reverse cholesterol transport in macrophages by controlling the expression of cholesterol transporters and apolipoproteins, including ABCA1, ABCG1, apoE, and apoC 154, 155.

## Autophagy of lipid homeostasis in DKD

Lipid and bioenergy are popular topics in DM research. Lipid metabolism plays an important role in the physiology and pathology of DKD. Autophagy is not only a cellular waste degradation pathway, but it is also a core way to maintain cell and organism homeostasis. Autophagy plays an important role in the pathogenesis of DKD 156. In the state of continuous hyperglycemia, multiple pathways and mechanisms will lead to the decrease of DKD autophagy activity, including lysosomal autophagy, mitochondrial autophagy, etc., also including podocyte and renal tubular cells of different kidney-resident parenchymal cells of autophagy 157, 158. Moreover, targeted improvement of autophagy is expected to be a new strategy for the treatment of DKD 159. Autophagy is involved in the regulation of lipid content. Decreased autophagy promotes lipid accumulation, inhibition of autophagy further increases lipid retention, and autophagy is further impaired as lipid content increases 160. Here, we focus on autophagy.

The autophagy pathway has an imperative role in physiology, with its primary function being to protect cells or organisms from starvation during nutrient deprivation by enabling them to recycle nutrients from digested organelles and macromolecules, as well as to ensure intracellular homeostasis through the removal of damaged organelles and abnormally folded proteins. Studies have shown that decreased autophagy promotes lipid accumulation, which in turn further inhibits autophagic function, thereby increasing lipid accumulation 160. Resveratrol can improve lipid metabolism in diabetic nephropathy rats through AMPKα/mTOR-mediated autophagy 161.

Lipids are increasingly involved in the control of biochemical processes and membrane remodeling underlying autophagosome biogenesis and autophagy more generally 162. Since autophagosome membranes largely lack transmembrane proteins, autophagosome biogenesis is thought to be largely regulated by lipid transfer and lipid modification as well as membrane-associated proteins 163. Lipids and lipid-metabolizing enzymes mediate the process of autophagy by controlling at least four fundamental aspects (**Table 1**). First, lipids mediate signaling. They regulate a signaling cascade that converges on the mTOR pathway, which in turn negatively regulates the initiation of autophagy. Central to this signaling cascade is class I PI3K and its product phosphatidylinositol-3,4,5-trisphosphate (PI(3,4,5)P3) 164. Second, lipids mediate the local recruitment of effectors to the membrane. Lipids act as local signals for membrane binding and control membrane dynamics by specifically recruiting cytoplasmic protein effectors that mediate membrane deformation, swelling, and vesicle trafficking. A typical example of this regulation is PI3P, which controls autophagosome biogenesis and maturation by this mechanism 165, 166. Third, lipids mediate covalent modification of proteins. Amine-containing phospholipids that include phosphatidylethanolamine covalently bind to members of the Atg8/LC3 family, providing a unique mode of regulation by anchoring these key factors stably to the membrane of phagocytes, mediating their extension and eventual closure 167. Fourth, lipids can control membrane dynamics independently of protein effectors by directly affecting the physicochemical properties of the lipid bilayer. Examples of such regulation include lipid rafts, cone-shaped lipids such as phosphatidic acid, which predisposes to or induces negative curvature, and cholesterol, which promotes or stabilizes the liquid-ordered phase within the bilayer 168. Cholesterol is associated with the organization of microdomains within the lysosomal membrane that control the effects of chaperone-mediated autophagy (CMA) and autophagosome-lysosome fusion 169, 170. Short-term cholesterol depletion leads to a rapid induction of autophagy, and the ER-localized cholesterol transporter GRAMD1C has been proposed as a negative regulator of starvation-induced macroautophagy/autophagy 163. Sphingolipids are ubiquitous membrane lipids in eukaryotes and are involved in the generation of a variety of membrane structures, including rafts, vesicles, and cytoplasmic vesicles. There are two major sphingolipids involved in autophagy, namely, ceramide and S1P 171. Exogenous application of short-chain ceramides, including C2-ceramides, stimulates autophagy, probably by promoting *de novo* synthesis of long-chain ceramides 172, 173. Long-chain ceramide partially activates autophagy by inhibiting the phosphorylation of Akt/protein kinase B (PKB) in the class I PI3K pathway, reducing mTOR activation and upregulating beclin1 function through JNK1-mediated dissociation of the beclin1-Bcl2 complex 172, 174; however, the specific mechanism underlying the role of sphingolipids in autophagy needs to be further elucidated. In sum, five major lipid classes are directly associated with autophagy (FAs, phospholipids, glycerides, sphingolipids, and sterols).

**Figure 3:**
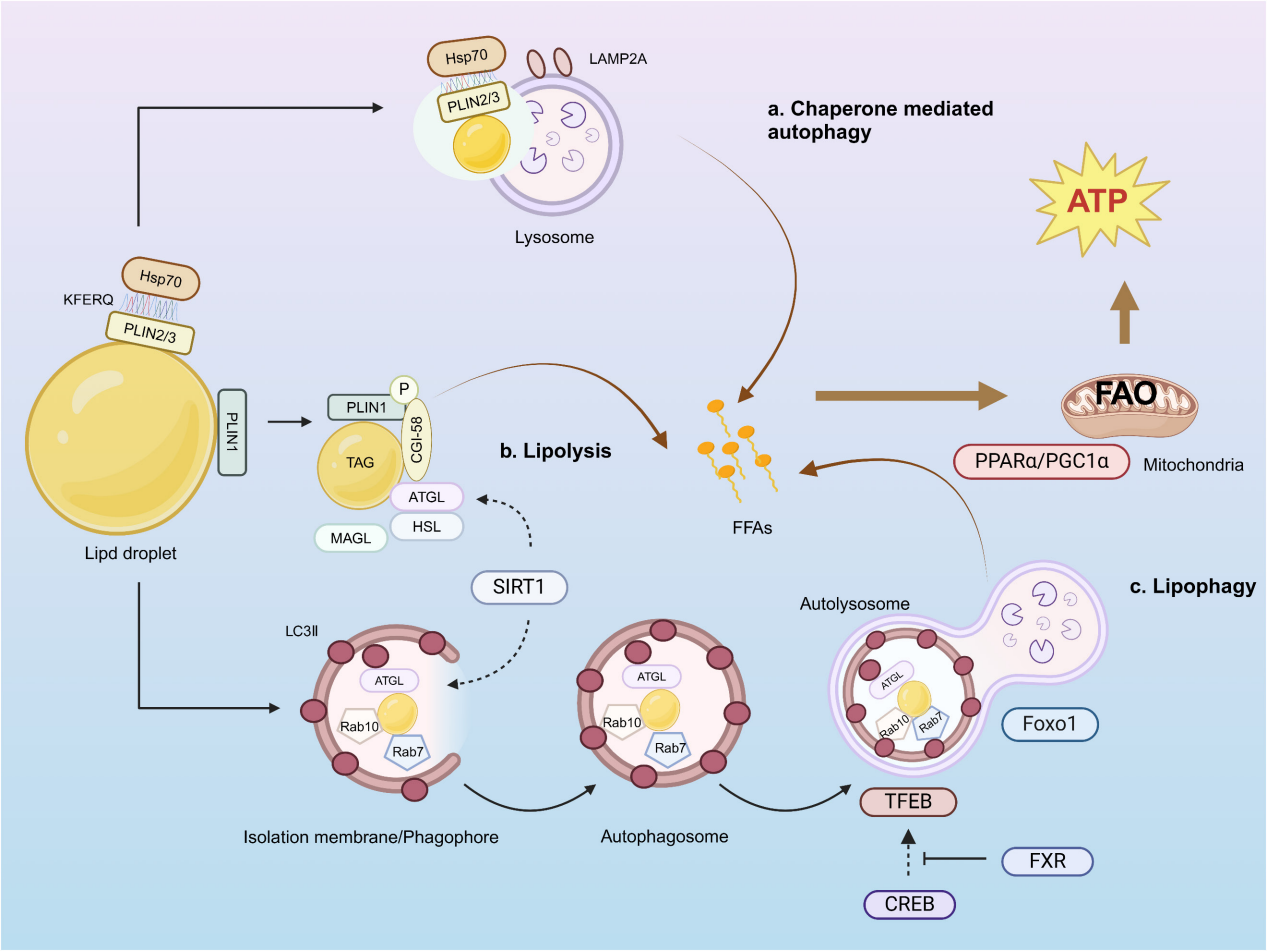
** Pathway of lipid droplet decomposition.** a) The KFERQ sequence in PLIN2/3 on the surface of LDs can bind to HSP70 and mediate chaperone-mediated autophagy. b) LDs can be decomposed by lipases ATGL, HSL, and MAGL. c) ATGL co-localizes with the autophagy marker protein LC3 to promote the formation of phagocytosed LDs and autophagosomes, while Rab7 on the surface of LDs promotes fusion between autophagosomes and lysosomes, leading to lipophagy occurrence. All three pathways generate FFAs and facilitate FAO for ATP production as an energy source.

Lipids themselves can also serve as substrates of autophagy, a pathway in which autophagic lysosomes directly consume cellular lipids in the form of LDs, a phenomenon known as lipophagy (**Fig. 3**), to achieve tissue energy homeostasis 175, 176. Synthesis of LDs occurs in the ER 177. Most cells produce LDs between 0.1 and 10 μm in size. LDs serve as cellular stores of neutral lipids, including triglycerides and cholesteryl esters, which contribute to the initiation of autophagy. The stored neutral lipids are mobilized during autophagy to support autophagic membrane formation 178. LDs are surrounded by phospholipid monolayers and LD shell proteins of the periplasmic lipoprotein family (PLIN) 179. The PLIN family consists of five members, PLIN1-5. PLIN1 and PLIN2 are located only on the surface of LDs and are degraded when not bound to LDs. PLIN3 and PLIN4 freely bind to or dissociate from LDs and remain stable even when released into the cytoplasm. PLIN5 is predominantly expressed in a number of highly oxidized tissues *in vivo*, including the heart, skeletal muscle, and liver. PLINs can participate in lipophagy by regulating the binding of lipases to LDs. Once the lipolysis signal is detected, PLIN1 rapidly phosphorylates and releases CGI-58, which ultimately activates ATGL and initiates lipolysis. PLIN2 and PLIN3 contain a CMA recognition sequence (KFERQ) that binds to a 70-kDa heat-shock protein (HSP70), thereby directing the LD toward the lysosome for CMA degradation 180. Cytosolic lipases mobilize LDs by disintegrating one triglyceride molecule into three FA molecules and one glycerol molecule through tandem reactions catalyzed by ATGL 181, HSL, and monoacyltriglyceride lipase (MAGL) 182. ATGL-deficient mice exhibit lipid accumulation in adipose tissue, heart, and liver 183. ATGL can co-localize with the autophagy marker protein LC3 on the surface of LDs and enhance the binding of LC3 to lysosomes and LDs, thereby enhancing lipophagy activity 184. Overexpression of HSL has been shown to reverse hepatic steatosis 185. The Rab GTPase protein family comprises key regulators of intracellular vesicle trafficking. Nearly 30 Rab family members have been identified on the surface of LDs, including Rab7, Rab10, Rab32, and Rab25. Among them, Rab7 is the most important protein 186. It can mediate the fusion of autophagosome membranes and late endocytic membranes 187. Mutations in Huntingtin lead to significant accumulation of LDs, suggesting that it plays an essential role in the regulation of lipophagy as an LD-recognizing receptor protein 188.

In addition, the occurrence of lipophagy is regulated by different mechanisms. ATGL-mediated signaling can promote autophagy/lipophagy through SIRT1 184. Starvation-induced activation of the transcription factor forkhead box protein O1 (FoxO1) regulates lipid content through transcriptional upregulation of LAL-mediated autophagy 189. Transcription factor EB (TFEB) plays a central role in lipid metabolism by regulating starvation-induced transcription through the PGC1-α-PPAR-α-lipophagy axis, which mediates lipid catabolism 190. cAMP response element-binding protein (CREB) upregulates autophagy genes, including Atg7, Ulk1, and TFEB, by recruiting the co-activator CRTC2 in the fasting state. In contrast, nutrients inhibit this effect by activating the nuclear receptor FXR 191. Starvation-induced PPAR-α activation reverses diet-induced inhibition of FXR-driven autophagy 192.

Blocking lipophagy promotes intracellular lipid accumulation, whereas activation of lipophagy leads to clearance of LDs. PNPLA5 is localized to LDs; the PNPLA5 pathway is the optimal pathway to initiate autophagy and is required for autophagy of multiple substrates, including degradation of autophagic junctions, bulk proteolysis, control of the number of mitochondria, and microbial clearance 178. The unique hydrophobic structural domains of ATG14 and the E2-like enzyme ATG3 were found to be key determinants in permitting surface recruitment of LDs and extending the autophagy machinery 193. Significant accumulation of LDs was accompanied by a significant reduction in FAO in hepatocytes treated with 3-methyladenine or by knockdown of Atg7 and Atg5^186^. Consistent with this, the accumulation of triglycerides in Atg5^-/-^ mouse embryonic fibroblasts also suggests that an autophagic defect hinders LD degradation 186. Decreased lipid autophagy and ectopic lipid deposition were observed in renal tubular cells of DKD patients, db/db mice, and HK-2 cells induced by high glucose 194. Shear stress can stimulate lipid autophagy, promote FA production, and promote mitochondrial ATP production through FAO to maintain renal metabolism 195.

## Conclusion and prospect

Currently, renal replacement therapy is the only available treatment modality for ESRD. Patients in whom DKD progresses to ESRD require substantial healthcare resources. Therefore, it is essential to provide early treatment to DKD patients. A growing body of evidence supports the essential role of lipid metabolism in the onset and progression of DKD. Here, we present the regulatory factors involved in the metabolism of different lipid types, including FAs, cholesterol, and sphingolipids, discuss the relationship between lipid metabolism and renal intrinsic cells, and emphasize the roles of targeted autophagy in the regulation of lipid homeostasis in DKD, in order to support the development of novel treatment strategies for DKD targeting lipid metabolism. It is of great value to identify new therapeutic targets and develop therapeutic drugs to improve lipid homeostasis and inhibit the progression of DKD. Numerous studies have shown that improving renal lipid deposition can reduce renal injury; however, the benefits of lipid-lowering therapy for DKD remain controversial. Clinical trials of commonly used lipid-lowering drugs, such as fenofibrate, statins, PCSK9 inhibitors, sodium-glucose transporter-2 inhibitors, glucagon-like peptide-1 receptor agonists, and mineralocorticoid receptor antagonists, have been far less aggressive than animal studies. The benefit may be limited to a reduction in proteinuria, and data on effects on renal lipotoxicity are lacking 84. Empagliflozin can improve FFA-induced renal tubular injury through the PPAR-γ/CD36 pathway 196. In a study of potential compounds to treat lipotoxicity in DKD, berberine has been shown to promote mitochondrial FAO in podocytes by activating PGC-1α 197. Tetrapeptide SS-31, which targets cardiolipin and protects the structure of mitochondrial cristae, protects kidney cells of C57 BL/6 mice after 28 weeks of high-fat diet, and prevents intracellular lipid accumulation 198. Synthetic S1p analogue FTY720 can alleviate S1P-induced podocyte injury by reducing inflammatory cytokines 199. Therefore, more systematic and comprehensive studies that focus on the role of lipid-lowering therapy in the treatment of DKD are necessary.

## Figures and Tables

**Table 1:** Lipids and lipid-metabolizing enzymes mediate the process of autophagy by controlling four fundamental aspects.

Mediation of autophagy by lipids	Molecules/Targets involved	Biological effects	References
Signaling	mTOR; PI3K; PI (3,4,5) P3	Negative regulation of autophagy	164
Local recruitment of effectors to the membrane	PI3P	Control of autophagosome biogenesis and maturation	165, 166
Covalent modification of proteins	phosphatidylethanolamine; Atg8/LC3	Extension and closure of the membrane	167
Membrane dynamics	Lipid rafts	/	168
	Phosphatidic acid	Induction of negative curvature	168
	Cholesterol	Promotion or stabilization of a liquid-ordered phase within a bilayer	168
		Control of chaperone-mediated autophagy	169, 170
		Control of autophagosome-lysosome fusion	169, 170
	GRAMD1C	Negative regulation of autophagy	163
	Short-chain ceramides	Stimulation of autophagy	172, 173
	Long-chain ceramide; JNK1; Beclin 1-Bcl2 complex; PI3K; mTOR	Activation of autophagy	172, 174
